# Quantitative Evaluation of Gastrorenal Shunt Morphology Using Three-Dimensional Computed Tomography Portography: Comparison With Intraoperative Venography

**DOI:** 10.7759/cureus.92351

**Published:** 2025-09-15

**Authors:** Yoshimi Fujii, Masato Tanikake, Yurie Nishimura, Kazuma Yasui

**Affiliations:** 1 Department of Diagnostic Radiology, Fujisawa City Hospital, Fujisawa, JPN

**Keywords:** 3dct-portography, brto, gastric varices, gastrointestinal bleeding, gastrorenal shunt, liver cirrhosis, portal hypertension

## Abstract

Background: Gastric varices are a serious complication of portal hypertension and may cause life-threatening bleeding when ruptured. Balloon-occluded retrograde transvenous obliteration (BRTO) is a standard endovascular treatment performed via the gastrorenal shunt (GRS). The present study examined concordance between three-dimensional computed tomography (3DCT) portography and intraoperative venography at the GRS outflow and evaluated the utility of 3DCT portography for preprocedural planning.

Methods: Nineteen patients who underwent BRTO between 2017 and 2024 were retrospectively analyzed. Preoperative 3DCT portography and intraoperative venography were used to assess the following parameters: the diameter of the stenosis, the diameter of the common trunk, the stenosis rate, the distance from the left renal vein to the stenosis, and the vertebral level of the stenosis. Paired t-tests, a Bland-Altman analysis, and correlation analysis were performed.

Results: No significant differences were observed in vessel diameters, the stenosis rate, or the distance from the left renal vein between the two modalities. The vertebral level of the stenosis was significantly more caudal on 3DCT portography (p = 0.0022). Bland-Altman plots showed good agreement, and all parameters showed moderate to strong correlations. In all cases, the stenosis was located just cranial to the confluence with the left adrenal vein, followed by a dorsal angulation averaging 62.9°, located approximately 20 mm cranial to the stenosis.

Conclusion: 3DCT portography showed high concordance with venography and consistently identified key anatomical features. It is a reliable tool for a preprocedural assessment in BRTO, aiding in risk stratification and individualized planning.

## Introduction

Gastric varices are a serious complication, occurring in approximately 20% of patients with portal hypertension [1]. Although they bleed less frequently than esophageal varices, rupture may lead to massive hemorrhage and high mortality [2,3]. Balloon-occluded retrograde transvenous obliteration (BRTO) is a well-established endovascular procedure for treating gastric varices. In this technique, the gastrorenal shunt (GRS), the primary outflow tract of gastric varices, is occluded using a balloon catheter, and a sclerosant is injected to induce thrombosis and obliterate the varices [4-6]. BRTO has a recurrence rate of <10% [7-9] and is widely regarded as an effective therapy for gastric varices [10-14].

A detailed understanding of the vascular anatomy and hemodynamics surrounding gastric varices is critical for successful BRTO. Gastric varices are primarily fed by the left gastric, posterior gastric, and short gastric veins and drain into the systemic circulation via the inferior phrenic, azygos, and periesophageal veins [15-18]. The left inferior phrenic vein is particularly important because its transverse branch drains into the inferior vena cava, while the descending branch connects with the left adrenal vein and then the left renal vein [19,20]. The dilation of this descending branch constitutes the GRS, a key portosystemic collateral in portal hypertension [16,18].

The GRS often exhibits a complex morphology, including stenosis, tortuosity, and the convergence of multiple small tributaries [18]. These features make deep retrograde catheterization during BRTO technically challenging. Three-dimensional computed tomography (3DCT) is widely utilized for evaluating the vascular anatomy and visualizing varices in patients with portal hypertension [21-24]. However, concordance between the findings of 3DCT portography and the intraoperative venographic anatomy has not yet been evaluated in detail. Therefore, the present study compared the two imaging modalities and identified distinctive morphological features on 3DCT portography, highlighting their clinical relevance.

This article was previously posted as a preprint on Research Square on April 11, 2025, under the title “Morphological Analysis of Gastrorenal Shunts Using Three-Dimensional Computed Tomography Portography: A Comparison with Intraoperative Venography During Balloon-Occluded Retrograde Transvenous Obliteration.”

## Materials and methods

Ethical considerations

This retrospective study was approved by the Ethics Committee of Fujisawa City Hospital (Approval No.: F2024027) and conducted in accordance with the Ethical Guidelines for Medical and Health Research Involving Human Subjects issued by the Ministry of Health, Labour and Welfare, the Ministry of Education, Culture, Sports, Science and Technology, and the Ministry of Economy, Trade and Industry of Japan. Written informed consent was not required due to the retrospective design; it was instead obtained through an opt-out process.

Patients

**Figure 1:**
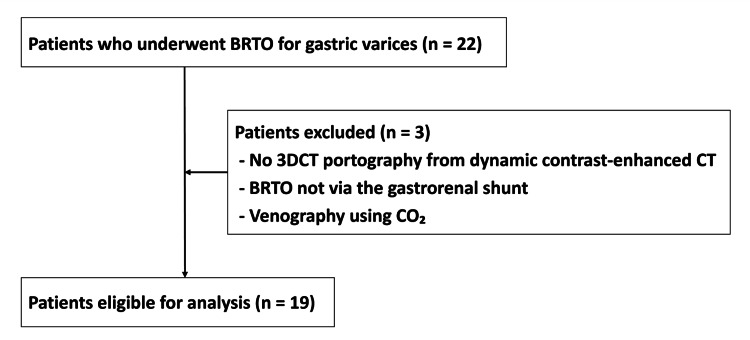
Flow chart of patient selection Between February 2017 and October 2024, 22 patients underwent BRTO for gastric varices at our institution. After excluding those without preprocedural dynamic contrast-enhanced CT, those not treated via the gastrorenal shunt, and those who underwent carbon dioxide venography, 19 patients were included in the final analysis. BRTO: balloon-occluded retrograde transvenous obliteration; 3DCT: three-dimensional computed tomography; CO_2_: carbon dioxide

We retrospectively analyzed 22 patients who underwent BRTO for gastric varices at our institution between February 2017 and October 2024. Exclusion criteria included the absence of 3DCT portography reconstructed from dynamic contrast-enhanced CT, BRTO performed via a non-GRS route, and intraoperative venography conducted with carbon dioxide. Consequently, 19 patients (14 men and five women; mean age, 66.1 ± 9.6 years) were included in the final analysis (Figure 1). Patient characteristics are summarized in Table 1.

**Table 1:** Patient characteristics BRTO: balloon-occluded retrograde transvenous obliteration; SD: standard deviation; BMI: body mass index; NASH: non-alcoholic steatohepatitis; HCC: hepatocellular carcinoma; PTO: percutaneous transhepatic obliteration; Location: Lg-f, fundus type; Lg-c, cardia type; Lg-cf, cardiofundal type; Form: F1, straight varices; F2, tortuous varices; F3, nodular varices; Color: Cw, white color varices; Cb, blue color varices

Characteristics	n = 19
Age (mean±SD, year)	66.1±9.6
Sex (male/female)	14/5
BMI (mean±SD, kg/m^2^)	27.1±4.2
Disease origin (virus/alcohol/NASH/other)	5/7/3/4
Child-Pugh class (A/B/C)	16/2/1
Child-Pugh score	5.8±1.4
Ascites (no/yes)	17/2
HCC (no/yes)	16/3
Esophageal varices (no/yes)	10/9
Hepatic encephalopathy (no/yes)	19/0
Portal vein thrombosis (no/yes)	18/1
History of gastric variceal rupture (no/yes)	14/5
History of treatment for gastric varices (no/yes)	14/5
History of endoscopic treatment (no/yes)	14/5
History of endovascular treatment (no/PTO/BRTO)	17/1/1
Endoscopic evaluation of gastric varices	
Location; Lg-f/Lg-c/Lg-cf	11/2/6
Form; F1/F2/F3	1/13/5
Color; Cw/Cb	7/12
Red-color sign (no/yes)	17/2
Mucosal findings (no/yes)	15/4
Bleeding findings (no/yes)	18/1

Among the 19 patients analyzed, five had a history of gastric variceal rupture treated with an endoscopic cyanoacrylate injection. One patient had undergone percutaneous transhepatic obliteration, and another had a history of BRTO. One patient experienced recurrence after both the endoscopic treatment and initial BRTO, requiring a second BRTO. These prior interventions were considered during the evaluation because of their potential effects on the vascular anatomy and imaging findings.

Preoperative contrast-enhanced CT and 3DCT portography

An iodinated contrast agent (600 mgI/kg) was administered intravenously at 3-3.5 mL/s. CT scans were acquired 45 seconds (arterial phase), 60 seconds (portal venous phase), and 90 seconds (equilibrium phase) post-injection. Imaging was performed using Somatom Definition Flash (Siemens Healthineers, Forchheim, Germany) or Aquilion One (Toshiba, Tokyo, Japan). All scans were obtained during a single inspiratory breath-hold. Axial images were reconstructed at a slice thickness of 1.0 mm and at 1.0-mm intervals using a standard soft-tissue convolution kernel. A dedicated workstation (Ziostation2, Amin, Tokyo, Japan) was used to generate 3DCT portography, enabling the visualization of gastric varices, the GRS, the left renal vein, the inferior vena cava, and vertebral structures.

Intraoperative venography

All procedures were performed using an interventional radiology (IVR)-CT system (Artis zee ceiling, Somatom Emotion6, Siemens Healthineers, Forchheim, Germany) under fluoroscopy at a pulse rate of four frames per second. In the BRTO procedure, we followed the technique described by Kanagawa et al. [4] (reproduced with permission from John Wiley & Sons). Briefly, an 8-Fr guiding sheath (Medikit, Tokyo, Japan) was inserted via the right femoral or internal jugular vein and advanced through the left renal vein to the GRS outflow tract. A 5.2-Fr balloon catheter (Terumo, Tokyo, Japan) with a balloon diameter of 13 or 20 mm was positioned beyond the confluence with the left adrenal vein into the GRS, and venography was performed.

Iodinated contrast medium (iomeprol; Iomeron 300, Bracco Imaging, Milan, Italy) was manually injected (5-7 mL) to obtain initial venographic images. The balloon catheter was further advanced into the GRS, and balloon-occluded venography was conducted. After confirming complete balloon occlusion of the GRS, 5% ethanolamine oleate mixed with iomeprol was injected into the gastric varices. If collateral veins were visualized under occlusion, coil embolization was performed beforehand to prevent reflux, followed by the injection of a sclerosant.

Measurement parameters

**Figure 2:**
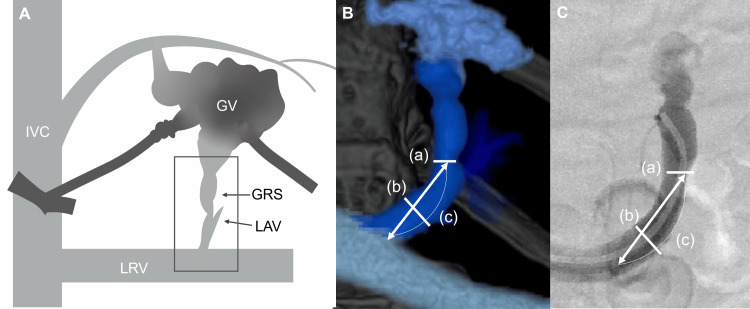
Morphological evaluation of the gastrorenal shunt outlet A: Schematic illustration showing the entire anatomy of gastric varices, including afferent veins, efferent veins, the GRS, left renal vein, and inferior vena cava. The GRS outlet is highlighted by a rectangle, indicating the region of interest in this study. B: 3DCT portography image showing measurement locations for the diameter of the stenosis (a), the diameter of the common trunk (b), and the distance from the left renal vein to the stenosis (c). C: Intraoperative venography image showing the corresponding measurement locations for parameters a, b, and c. GV: gastric varices; GRS: gastrorenal shunt; IVC: inferior vena cava; LAV: left adrenal vein; LRV: left renal vein Image Credit: Yoshimi Fujii

**Figure 3:**
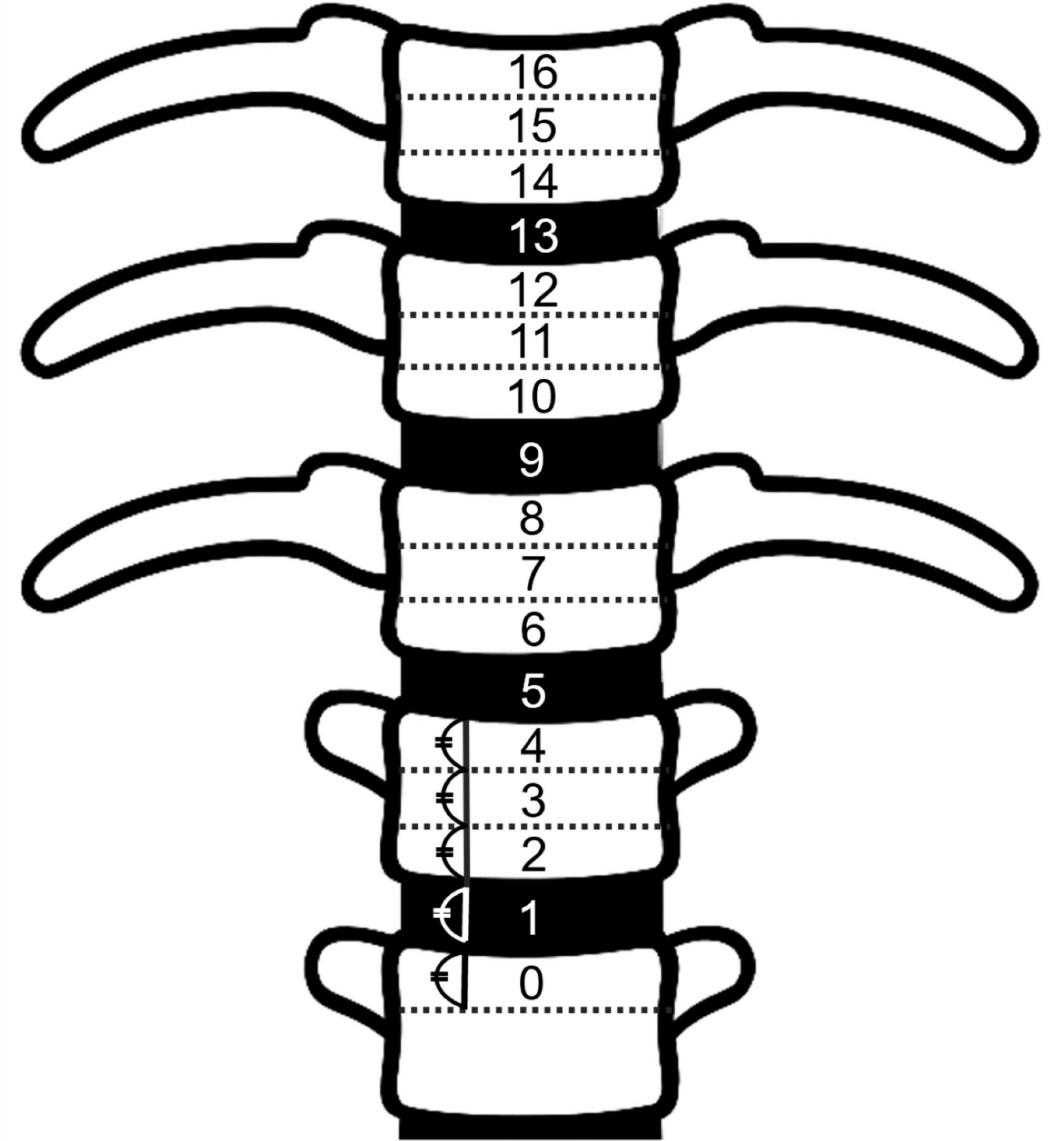
Vertebral level classification for the anatomical localization of the stenosis The thoracolumbar spine from T10 to L2 is labeled from level 16 (upper edge of T10) to level 0 (upper edge of L2). Each vertebral body and intervertebral disc is further subdivided into the upper, middle, and lower thirds, with intervertebral discs treated as separate anatomical levels. Image Credit: Yoshimi Fujii

We measured the diameter of the stenosis (a), the diameter of the common trunk (b), the stenosis rate calculated as $(1 - \tfrac{a}{b}) \times 100\%$, the distance from the left renal vein to the nearest stenosis (c), and the vertebral level of stenosis using both 3DCT portography and intraoperative venography (Figure 2). Vertebral levels were divided into upper, middle, and lower thirds, with intervertebral discs considered separate anatomical levels. Levels were labeled from the upper edge of L2 (defined as 0) to T10 (Figure 3).

Measurements from 3DCT portography were performed using volume-rendered (VR) images and independently assessed by a board-certified radiologist. Using 3DCT portography alone, we further evaluated the cranial morphology beyond the first stenosis, including the angle of the most prominent dorsal angulation and the distance from the stenosis to the angulation point. The angulation angle was measured on VR images viewed from a 90° left or right lateral perspective to clearly depict dorsal deviations.

Statistical analysis

To assess concordance between 3DCT portography and intraoperative venography, we performed paired t-tests for normally distributed data and Wilcoxon signed-rank tests for non-parametric data. A Bland-Altman analysis was conducted using scatter plots of the mean versus the difference in paired values to assess fixed or proportional bias. Limits of agreement were defined as the mean difference ± 1.96 × the standard deviation. A correlation analysis was performed using Pearson’s or Spearman’s coefficients, depending on data distribution, to assess the strength of linear relationships between the two modalities. All statistical analyses were conducted using Modified R Commander (version 4.4.2, developed and distributed by the Laboratory of Eiki Tsushima, Hirosaki University, Japan), with significance set at p < 0.05.

## Results

**Figure 4:**
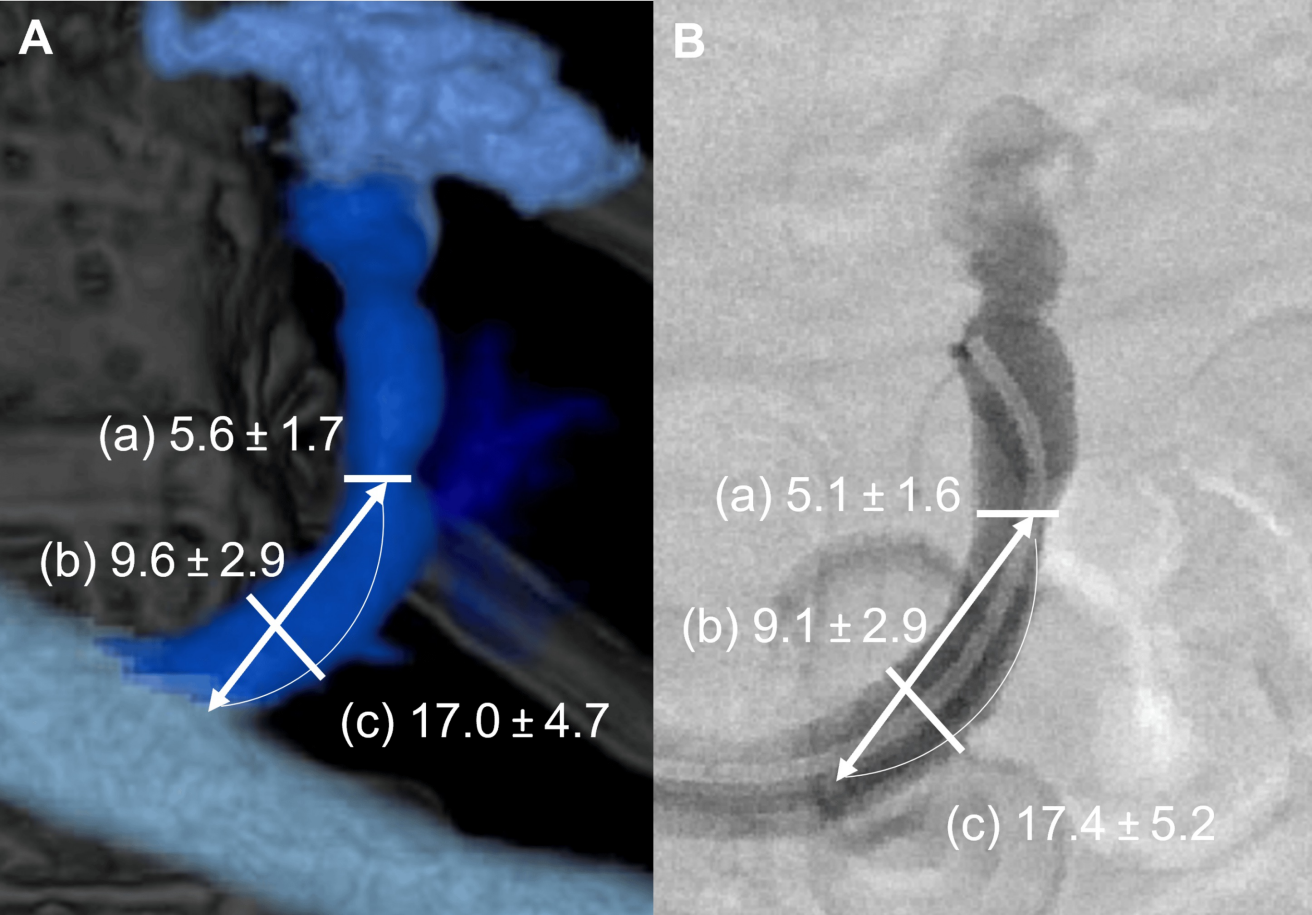
Measured values of the gastrorenal shunt outlet morphology A: 3DCT portography image with annotated measurements (in mm), including the diameter of the stenosis (a), the diameter of the common trunk (b), and the distance from the left renal vein to the stenosis (c). B: Intraoperative venography image showing the corresponding measurements of parameters a, b, and c (in mm). 3DCT: three-dimensional computed tomography

**Figure 5:**
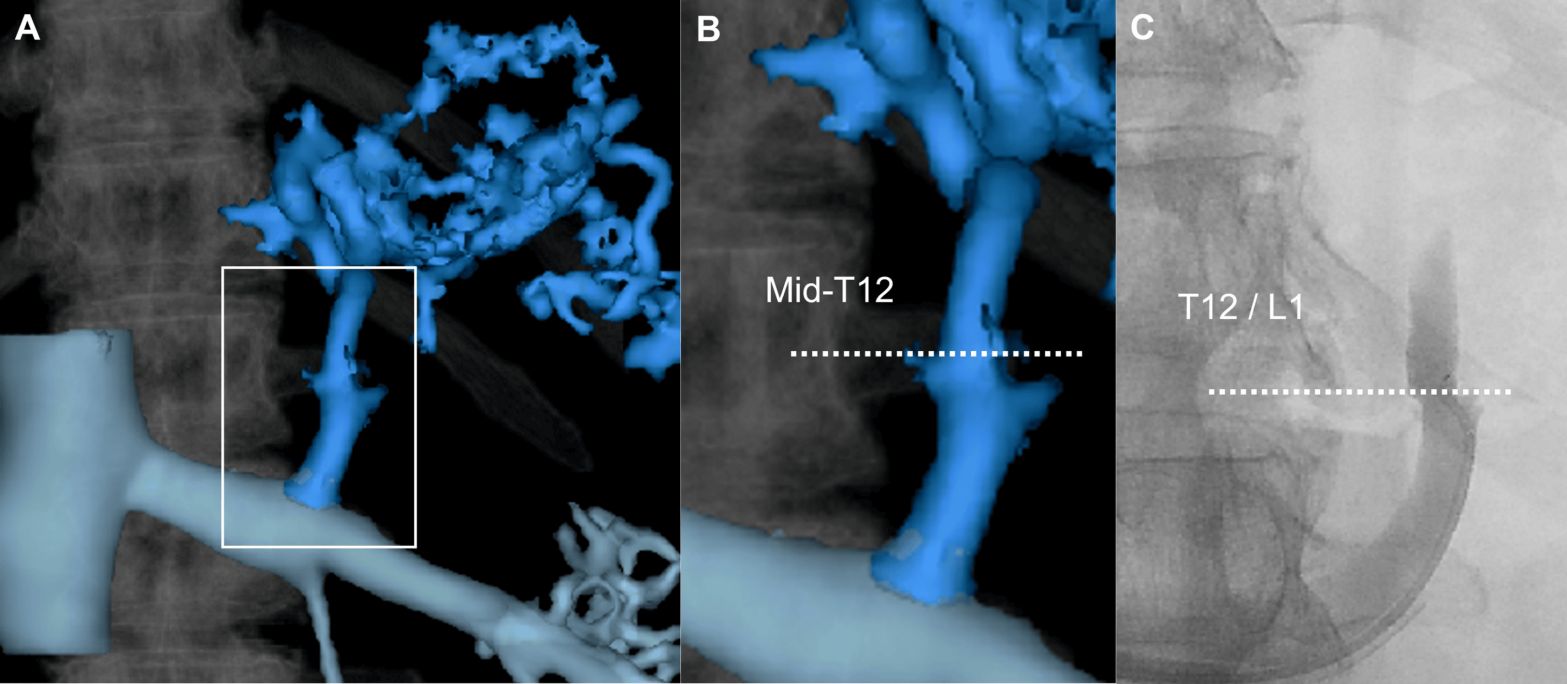
Discrepancy in the vertebral level of the stenosis between 3DCT portography and intraoperative venography A: 3DCT portography image of gastric varices, including the GRS, left renal vein, inferior vena cava, and vertebral bodies. The GRS outlet is highlighted by a rectangle to indicate the region of interest. B: 3DCT portography image showing the stenosis of the GRS outlet located at the mid-T12 vertebral level. C: Intraoperative venography image showing the corresponding stenosis at the T12/L1 intervertebral level. GRS: gastrorenal shunt; 3DCT: three-dimensional computed tomography

The paired statistical analysis showed no significant differences in the distance from the left renal vein to stenosis, the diameter of stenosis, the diameter of the common trunk, or the stenosis rate between 3DCT portography and intraoperative venography. However, the vertebral level of stenosis was significantly more caudal on 3DCT portography than on intraoperative venography (1.8 ± 1.1 vs. 2.6 ± 0.9; p = 0.0022), with a mean difference of 0.79 vertebral levels (Table 2, Figures 4, 5).

**Table 2:** Comparison of measurements between 3DCT portography and gastrorenal shunt venography 3DCT: three-dimensional computed tomography; GRS: gastrorenal shunt; LRV: left renal vein

Parameter	3DCT Portography	GRS Venography	p-value
Diameter of stenosis (mm)	5.6±1.7	5.1±1.6	0.056
Diameter of common trunk (mm)	9.6±2.9	9.1±2.9	0.166
Stenosis rate (%)	40.9±13.9	43.0±11.5	0.399
Distance from LRV to the stenosis (mm)	17.0±4.7	17.4±5.2	0.303
Vertebral level of the stenosis	1.8±1.1	2.6±0.9	0.0022
-	upper L1 to mid L2	lower T12 to upper L1	-

**Figure 6:**
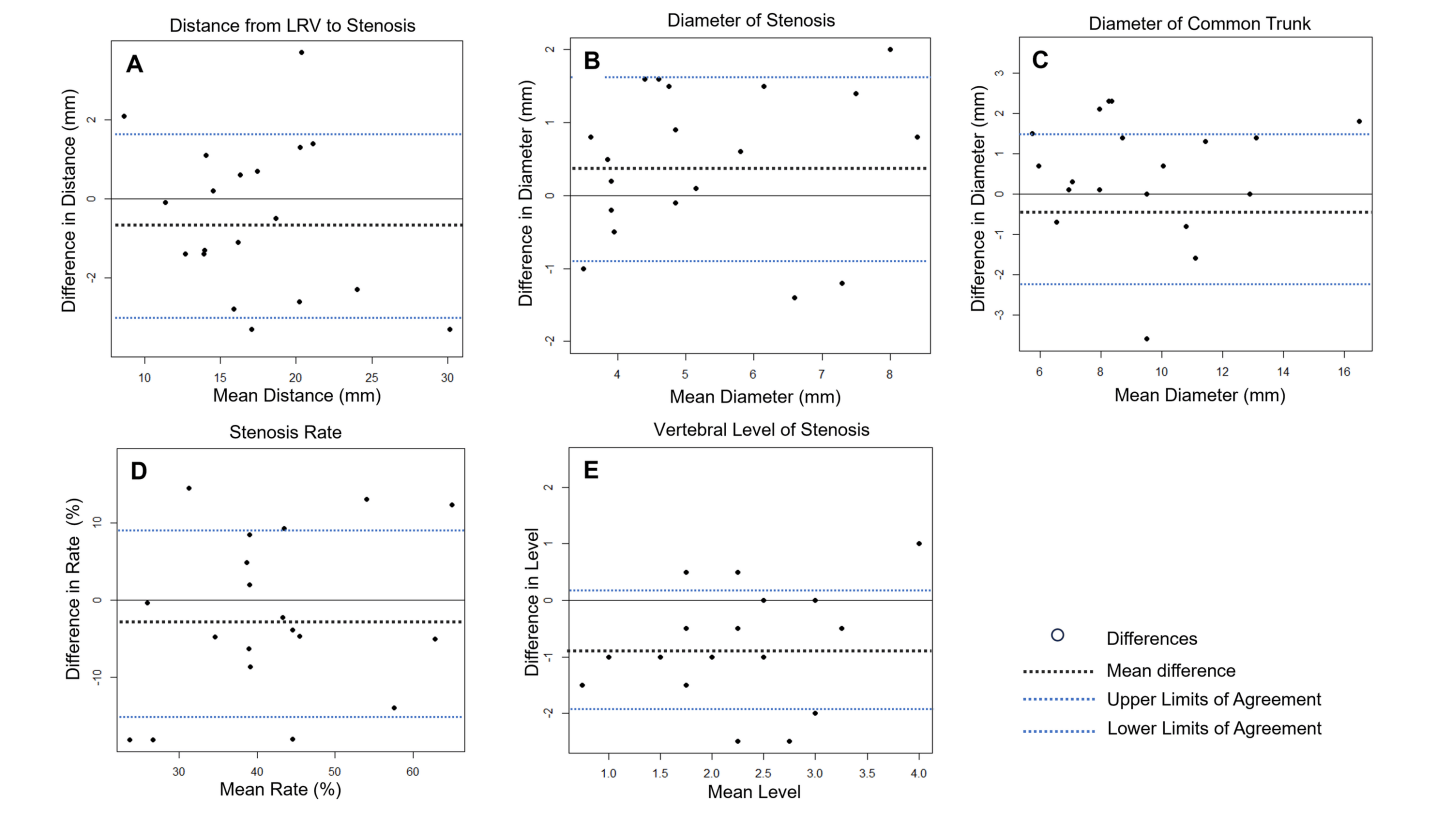
Bland-Altman plots comparing 3DCT portography and intraoperative venography measurements A: distance from LRV to the stenosis; B: diameter of the stenosis; C: diameter of the common trunk; D: stenosis rate; E: vertebral level of the stenosis Plots show the difference between paired measurements (y-axis) against their mean (x-axis). Good agreement was observed for parameters A–D, with no evidence of a fixed or proportional bias. In contrast, panel E shows a significant fixed bias in vertebral level measurements, with a 95% confidence interval for the mean difference ranging from –1.25 to –0.33. LRV: left renal vein; 3DCT: three-dimensional computed tomography

The Bland-Altman analysis showed strong agreement for the distance to the stenosis, the diameter of the stenosis, the diameter of the common trunk, and the stenosis rate, with no fixed or proportional bias. In contrast, vertebral levels showed a significant fixed bias, with a 95% confidence interval of -1.25 to -0.33 (Figure 6).

**Figure 7:**
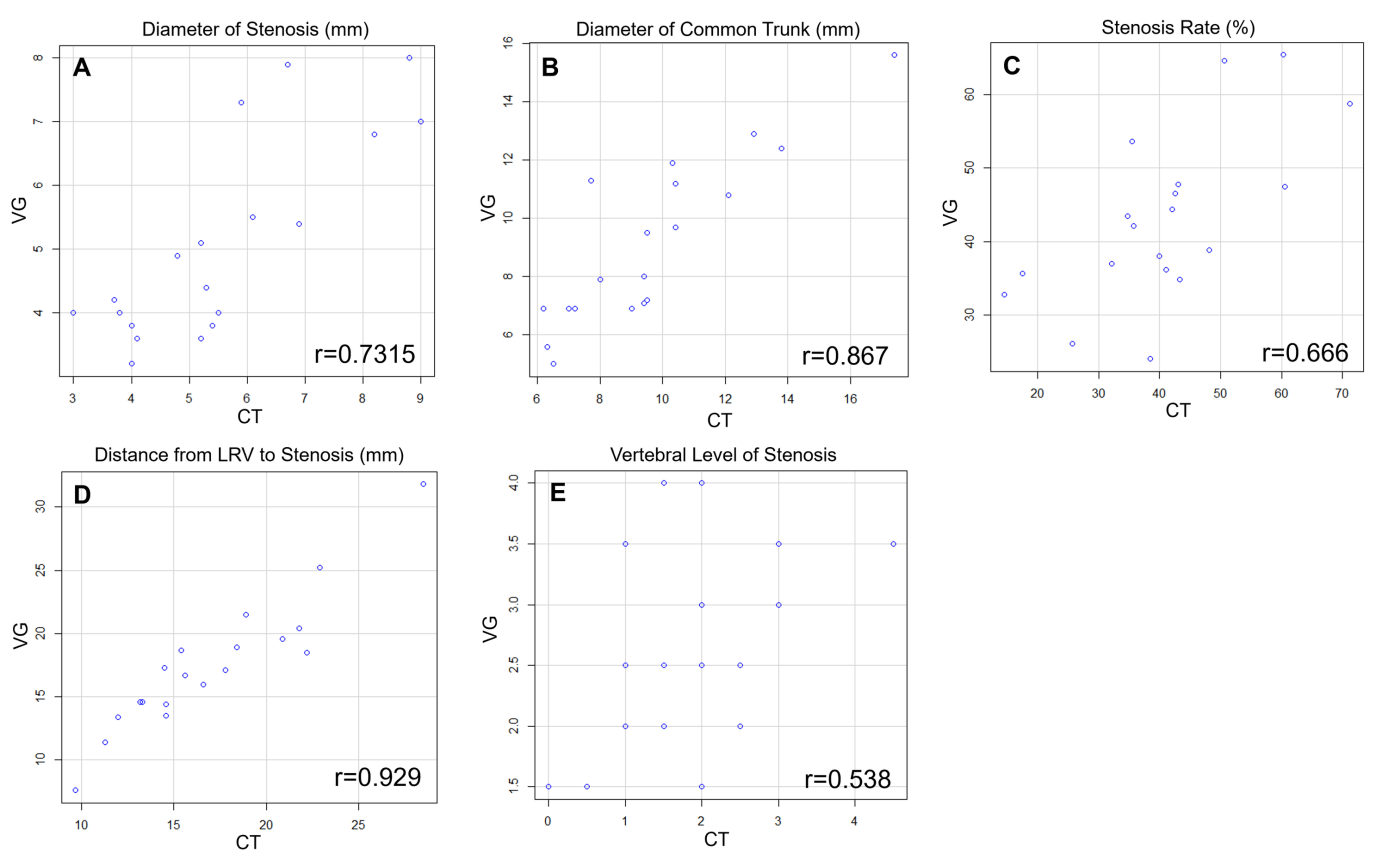
Scatter plots showing correlations between measurements obtained by 3DCT portography and intraoperative venography A: distance from LRV to the stenosis; B: diameter of the stenosis; C: diameter of the common trunk; D: stenosis rate; E: vertebral level of the stenosis Each plot shows the relationship between paired values measured by the two modalities. The correlation analysis revealed moderate to very strong correlations for all five parameters. LRV: left renal vein; 3DCT: three-dimensional computed tomography

The correlation analysis revealed moderate to very strong correlations for all five parameters (Figure 7).

**Figure 8:**
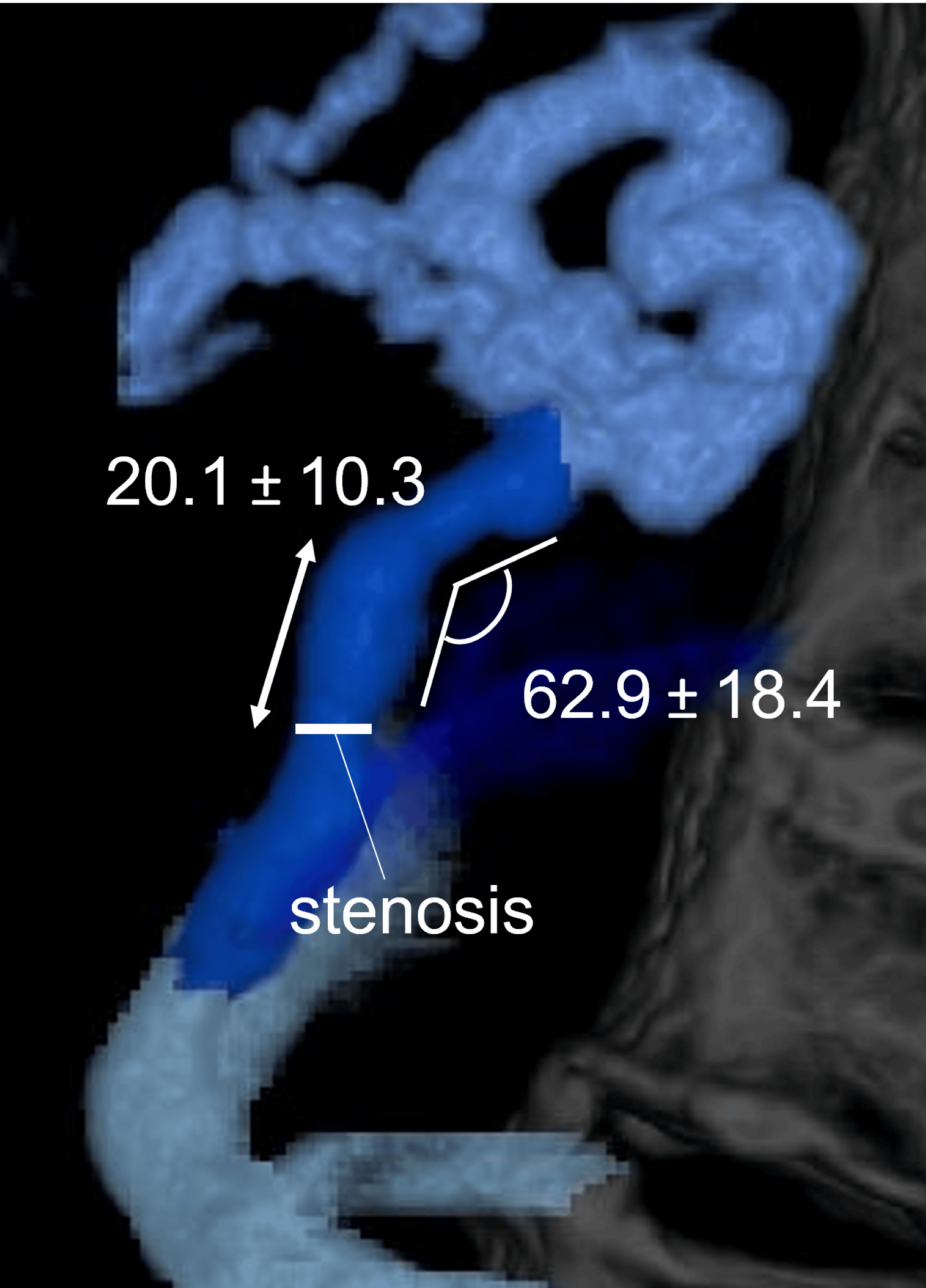
Measured values of the gastrorenal shunt outlet morphology Sagittal view of 3DCT portography obtained from a 90° left lateral perspective, illustrating the measurement of the dorsal angulation (in degrees) and the distance from the stenosis to the angulation point (in mm). 3DCT: three-dimensional computed tomography

The morphological assessment using 3DCT portography revealed that the stenosis was located just cranial to the confluence with the left adrenal vein in all 19 cases. A prominent dorsal angulation, averaging 62.9 ± 18.4°, was observed at a mean distance of 20.1 ± 10.3 mm cranial to the stenosis (Figure 8).

## Discussion

Although 3DCT portography is widely used to non-invasively assess the portal venous anatomy and hemodynamics prior to gastric variceal treatment, its morphological concordance with intraoperative venography has not yet been fully investigated. In the present study, we quantitatively assessed the outflow tract of the GRS using 3DCT portography and compared the results obtained with intraoperative venography. Concordance was high for parameters such as the distance from the left renal vein to the stenosis and vessel diameter.

Since the early 2000s, advances in multi-detector CT have improved the depiction of gastric varices [25-27]. Matsumoto et al. reported that three-dimensional portography enabled the high-resolution visualization of fundal varices and the GRS, supporting its utility as a non-invasive preoperative tool for BRTO [28]. Similarly, Kiyosue et al. emphasized the importance of identifying inflow and outflow veins using contrast-enhanced CT, which provides essential information for treatment planning [17]. While previous studies focused on the overall hemodynamics of gastric varices, the novelty of the present study lies in its quantitative evaluation of the outflow tract morphology, an anatomical feature that directly affects catheter manipulations during BRTO.

In all cases, the stenosis was located just cranial to the common trunk of the left adrenal vein. Additionally, a prominent dorsal angulation averaging 62.9 ± 18.4° was observed at a mean distance of 20.1 ± 10.3 mm cranial to the stenosis. Saad et al. were the first to describe GRS outlet stenosis, noting web-like narrowing at the junction of the GRS proper and the left adrenal vein [18,29]. The present results provide the quantitative confirmation of their observations.

Interventional radiologists are empirically aware that catheter manipulations within the GRS are associated with a risk of venous injury. This vulnerability stems from the anatomical characteristics of the GRS. Venous walls are thinner than arterial walls and lack substantial muscular and elastic layers, making them more susceptible to external force. Additionally, since the catheter is introduced in a retrograde manner, the course of the vessel is often unclear without balloon occlusion, which makes it difficult to identify anatomical landmarks. Furthermore, as demonstrated herein, the presence of angulation immediately distal to the stenosis may concentrate the forward force required to traverse the stenosis on the inner wall of the angulated segment, potentially contributing to vessel injury.

Although most retroperitoneal hemorrhages resolve spontaneously, intraperitoneal bleeding may occur in cases with vascular anomalies and become life-threatening [30]. This underscores the critical importance of a thorough preprocedural risk assessment. 3DCT portography allows for the precise identification of high-risk features prior to the procedure. When advancing a catheter beyond the stenosis into the deeper portion of the GRS, careful device selection, such as using a microcatheter or microguidewire, is essential to minimize the risk of venous injury at angulated segments.

A significant difference was observed in the vertebral level of stenosis, with 3DCT portography depicting the stenosis at a more caudal level than intraoperative venography. This discrepancy may be attributed to differences in the respiratory phase: preoperative CT was performed during inspiration, whereas intraoperative venography was conducted during expiration. Despite this discrepancy, no significant difference was found in vessel diameters or in the distance from the left renal vein to the stenosis between the two modalities. One possible explanation is that the stenotic segment represents a structurally fixed lesion, such as fibrotic narrowing or venous valves, which are less affected by physiological changes in intrathoracic or intra-abdominal pressure. Additionally, contrast injections during both CT and venography may have a standardized intravascular pressure, minimizing respiratory phase-related variations. Although respiratory motion may shift the absolute position of a vessel relative to the spine, it does not necessarily change the vessel’s internal architecture or relative geometry. These factors may explain the observed consistency in diameter and length measurements.

This study has several limitations that need to be addressed. This was a single-center retrospective study with a small sample size. Although the sample size was sufficient for statistical analysis, the relatively small cohort may limit the generalizability of our conclusions. In addition, intraoperative venography was manually performed, which may have introduced variability in contrast injection volume and speed, potentially affecting image quality and consistency. Another limitation is that interobserver agreement was not assessed for vessel diameter or angulation measurements, introducing potential subjectivity. Finally, the present study focused solely on morphological assessment, without examining the impact of stenosis or angulation on technical difficulty, complication risks, or treatment efficacy during BRTO; thus, the clinical effectiveness of these parameters remains uncertain. Further research is needed to clarify these relationships and whether a preoperative morphological assessment contributes to risk stratification and procedural planning.

## Conclusions

The present study demonstrated that 3DCT portography is a reliable tool for quantitatively assessing the outflow tract of the GRS, showing strong concordance with intraoperative venography in key parameters, such as the stenosis diameter, stenosis rate, and the distance from the left renal vein to the stenosis. Preoperative CT also consistently identified critical morphological features, including the stenosis and dorsal angulation, which affect catheter manipulations and may help reduce procedural risks.

Collectively, these results support the utility of 3DCT portography not only as a diagnostic modality but also as a foundation for individualized procedural planning in BRTO. Further studies are warranted to clarify the relationships between these morphological features and procedural complexity or clinical outcomes.
